# Calcium Lactate Amendment Improves Saline Soil Properties and Enhances Pepper Resistance Under Short-Term Waterlogging Conditions

**DOI:** 10.3390/plants15152268

**Published:** 2026-07-24

**Authors:** Yuquan Lin, Shuwen Hu

**Affiliations:** 1 College of Resources and Environment Sciences, China Agricultural University, No. 2 Yuanmingyuan West Road, Beijing 100193, China; 15077122203@163.com; 2 China Beijing Key Laboratory of Farmland Soil Pollution Prevention-Control and Remediation, College of Resources and Environmental Sciences, China Agricultural University, No. 2 Yuanmingyuan West Road, Beijing 100193, China

**Keywords:** calcium lactate, soil amendment, saline soil, pepper, crop quality

## Abstract

Saline soils subjected to short-term waterlogging are a constraint on the sustainable production of protected vegetables, triggering soil structural degradation, salt accumulation, and oxidative damage in plants, which seriously damages crop yield and quality. Calcium-based amendments have attracted considerable attention for improving soil conditions and enhancing plant stress tolerance. However, the effects of calcium lactate amendment on saline soil improvement and pepper physiological responses under short-term waterlogging conditions remain insufficiently understood. Different application rates of calcium lactate were applied to saline pepper croplands under waterlogged conditions. The effects on soil physicochemical properties, nutrient availability, plant antioxidant physiology, fruit yield, and quality were analyzed. Calcium lactate improved the physicochemical properties of waterlogged soil and increased the available nutrients in the soil, with the content of available phosphorus and available potassium increasing by 565–855% and 1.60–35.54%, respectively. It enhanced the activities of superoxide dismutase, peroxidase, and catalase, reduced malondialdehyde accumulation (up to 39% compared with CK), and increased proline content (up to 54.62% compared with CK), thereby promoting plant physiological adaptation under short-term waterlogging conditions and improving pepper yield and fruit quality. Higher doses of calcium lactate exhibit better ameliorative effects. Correlation analysis and partial least squares path modeling suggested that calcium lactate application was associated with improved pepper yield and quality through pathways related to soil improvement and plant physiological regulation. This study provides insights into the potential application of calcium lactate for saline soil improvement and sustainable pepper production under short-term waterlogging conditions.

## 1. Introduction

With the intensification of protected agriculture, soil salinization has become a critical constraint on sustainable vegetable production, while the increasing frequency of short-term waterlogging events further exacerbates growth limitations of crops cultivated in saline soils. Soil salinization is a land degradation process caused by excessive accumulation of soluble salts in soil, which deteriorates soil physicochemical properties, disrupts ionic balance, and reduces nutrient availability, thereby inhibiting normal crop growth [1,2]. Salt accumulation can disrupt soil aggregate structure, decrease soil porosity, and reduce water infiltration capacity, thereby increasing the risk of waterlogging under short-term rainfall or excessive irrigation conditions. Short-term waterlogging caused by excessive rainfall or irrigation can further alter the rhizosphere oxygen environment and nutrient migration processes, disrupt soil aggregate stability, reduce soil aeration, and induce anaerobic conditions. These anaerobic conditions promote the reduction of iron, manganese, and other elements to toxic forms and facilitate the accumulation of reducing agents, such as organic acids and hydrogen sulfide [3]. Consequently, root respiration and nutrient absorption are inhibited, ultimately giving rise to hindered crop growth and reduced yield. Previous studies have demonstrated that waterlogging disasters have become a major abiotic stressor affecting crop yield and quality in global agricultural production. Waterlogging and soil salinization caused by improper irrigation will significantly reduce the production potential of the agricultural system, and the economic loss in some regions can exceed 30% [4].

Pepper is one of the most economically important vegetable crops in China, and its planting area has been stable at over 2 million hm^2^ for a long time. As one of the vegetables with the largest planting area in China [5], it plays an important role in human nutrition, food processing, and rural economic development [6]. With the rapid development of protected agriculture, pepper production has increasingly concentrated in protected cultivation systems, including greenhouses and plastic tunnels. In protected cultivation systems, frequent irrigation, continuous soil utilization, and limited drainage conditions often result in persistent accumulation of soil salts and water, thereby increasing the risk of short-term waterlogging events [7]. In addition, the root system of pepper is shallow and extremely sensitive to soil hypoxia. Waterlogging conditions will lead to slow growth of pepper plants, falling flowers and fruits, and increased disease occurrence, which triggers a significant decrease in yield and quality [8]. Therefore, investigating the growth and physiological responses of pepper under short-term waterlogging conditions in saline soils and identifying effective agricultural management strategies are essential for alleviating waterlogging stress and improving pepper yield and quality.

Applying soil amendments is an important agronomic practice for improving the physicochemical properties of saline soils and alleviating waterlogging stress. In recent years, increasing evidence has demonstrated that different functional materials can effectively promote plant growth and enhance stress tolerance by improving soil environments and regulating plant physiological processes [9]. However, in saline agricultural soils, salt accumulation is often accompanied by soil structural degradation and restricted water–salt transport, while short-term waterlogging further reduces oxygen supply in the root zone and intensifies ionic imbalance and oxidative stress. Moreover, soil water saturation restricts oxygen diffusion, resulting in alterations in redox conditions [10], which further affect soil ion migration, nutrient availability, and the stability of the root-zone environment [11].

The application of soil amendments is an effective agronomic strategy to alleviate waterlogging stress. Calcium-based amendments have attracted extensive attention because they have both soil structure regulation and plant stress resistance promotion effects [12,13]. From the perspective of soil physics, calcium ions (Ca^2+^) can promote the flocculation of clay particles and organic matter particles through “cation bridging”, and enhance the formation and stability of aggregates [14,15]. Furthermore, Ca^2+^ can replace Na^+^ adsorbed on the surface of soil colloids, thereby inhibiting clay dispersion, improving soil aggregate structure, and promoting salt leaching [16,17]. From the aspect of plant physiology, calcium is crucial in maintaining the cell wall, cell membrane structure, cell membrane structural stability and the signal transduction process [18]. Exogenous calcium can boost the activity of antioxidant enzymes and improve the stress resistance of plants. In recent years, research on calcium-based amendments in waterlogged soil has made remarkable progress at home and abroad. Traditional calcium-based amendments, including lime, dolomite, and gypsum, have been widely applied for saline–alkali soil improvement due to their ability to provide Ca^2+^ and regulate soil chemical properties [16,19]. Carbonate-based materials have advantages such as wide availability, low cost, and the ability to regulate soil acid–base conditions. However, their effectiveness mainly depends on carbonate mineral transformation processes, which are restricted under highly alkaline conditions, and their effects on regulating active carbonate pools and promoting soil organic carbon accumulation are limited [20]. Dolomite can simultaneously provide Ca^2+^ and Mg^2+^, which contributes to improving soil Ca/Mg balance and plant nutrient supply. However, the release of Ca^2+^ and Mg^2+^ from dolomite is controlled by mineral dissolution processes and soil environmental conditions, which may limit its effectiveness in rapidly regulating saline–alkali stress [21,22]. Gypsum can effectively provide soluble Ca^2+^ and promote Na^+^ exchange and leaching, but its application is accompanied by SO_4_^2−^ input, which may alter soil anion composition [23,24]. Lime and gypsum-based remediation technologies have been widely applied in China and other saline–alkali agricultural regions, forming relatively mature theories and practical systems for saline–alkali soil improvement [25,26,27]. In addition to improving soil environments, the regulatory effects of calcium-based materials on plant stress tolerance have gradually attracted increasing attention. Wang et al. [28] insisted that fertilization with novel calcium peroxide (CaO_2_) can reduce the anaerobic intensity of rapeseed roots and enhance their resistance to waterlogging. Shalaby and Ramadan et al. [18] found that calcium treatment can not only improve plant antioxidant defense and reduce cell damage caused by reactive oxygen species accumulation under adverse conditions, but also promote plant growth and nutrient absorption, enhance photosynthetic capacity and biomass accumulation, and significantly increase pepper yield. As a highly soluble and environmentally friendly organic calcium salt, calcium lactate can slowly release Ca^2+^, and its lactate radical can be used by soil microorganisms as an organic carbon source, which is beneficial to improving the rhizosphere microecological environment. Previous studies have shown that calcium lactate can promote soil colloidal flocculation and aggregate formation, and improve soil structural stability [16]. However, studies investigating the effects of calcium lactate on pepper growth and physiological responses under short-term waterlogging conditions in saline soils remain limited. Therefore, investigating the application of calcium lactate for saline soil improvement and its role in enhancing the physiological resistance of pepper to waterlogging stress can provide a theoretical basis for the remediation of saline-protected vegetable fields and high-quality, efficient pepper production.

In this study, a field experiment was conducted in saline pepper croplands with different application rates of calcium lactate to systematically investigate its effects on soil physicochemical properties, pepper growth, physiological characteristics, yield, and quality. The objective of this study was to elucidate the mechanisms by which calcium lactate regulates the saline soil environment and affects the short-term waterlogging responses of pepper, thereby providing a theoretical basis for the scientific improvement of saline-protected vegetable fields and stress-resistant cultivation of pepper.

## 2. Results

### 2.1. Dose Effects of Calcium Lactate Amendment on Physicochemical Properties of Soil Foundation in Each Soil Layer

**Figure 1:**
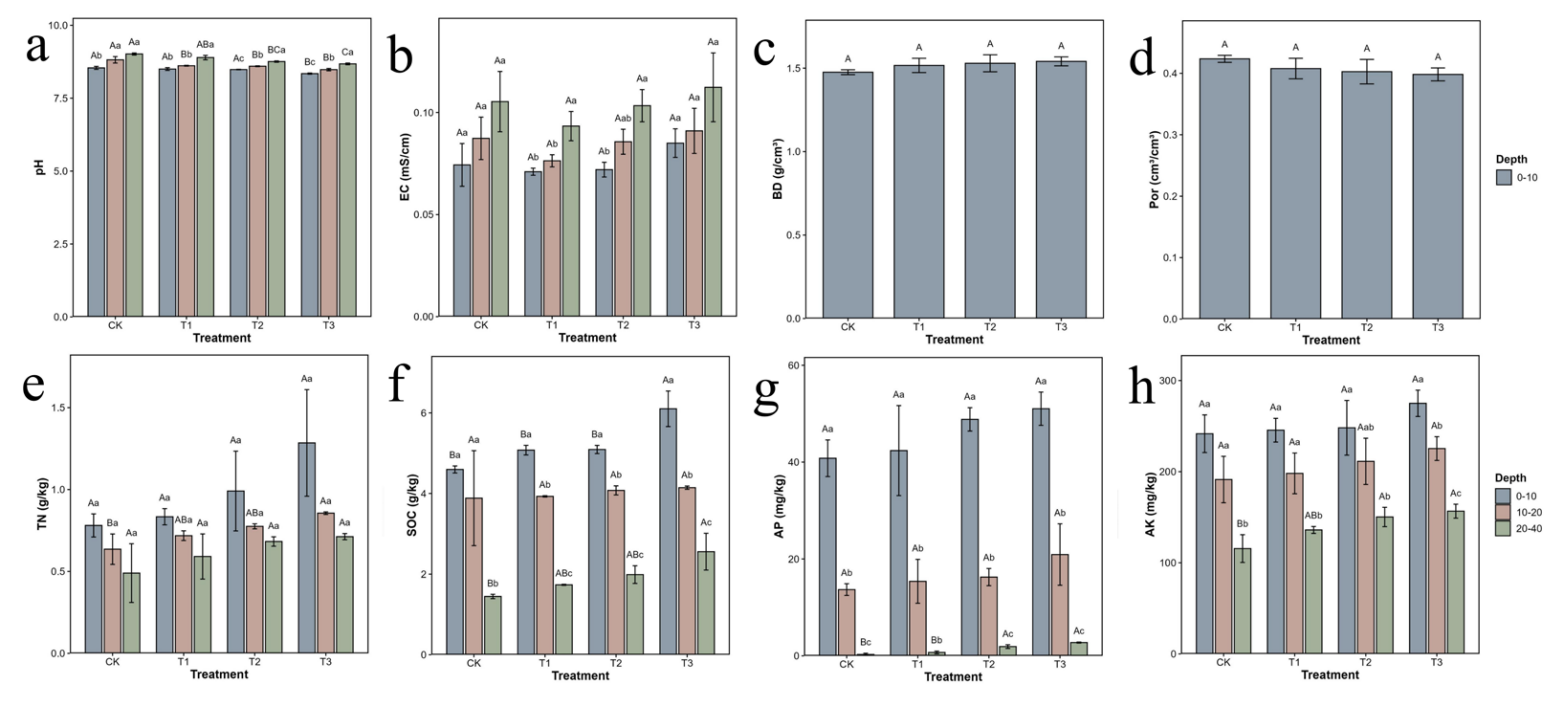
Effects of different dosages of calcium lactate amendment on the physicochemical properties of soil foundation in different soil layers. (**a**) pH; (**b**) electrical conductivity (EC); (**c**) bulk density (BD); (**d**) porosity (Por); (**e**) total nitrogen (TN); (**f**) soil organic carbon (SOC); (**g**) available phosphorus (AP); (**h**) available potassium (AK). CK, T1, T2, and T3 represent 0, low-dose, medium-dose, and high-dose calcium lactate application rate treatments, respectively. 0–10, 10–20, and 20–40 cm refer to various soil layer depths, respectively. Data are mean ± SD (*n* = 3). Various capital letters indicate significant differences between different treatments for the same index (*p* < 0.05), and different lowercase letters denote significant differences between different soil layers for the same treatment (*p* < 0.05).

According to the results, calcium lactate application dramatically improved soil physicochemical properties in different soil layers with an apparent dose effect. Figure 1 indicates that with the increasing calcium lactate application, soil pH and bulk density generally decreased, and soil porosity and nutrient content increased significantly. However, there was no significant difference in EC. Specifically, the decrease and increase in T3 treatment were the most apparent, and the pH of each soil layer decreased by 2.36–3.92% compared with CK (*p* < 0.05). The bulk density increased slightly and the porosity decreased slightly in the 0–10 cm soil layer without significant differences between treatments. Meanwhile, the contents of total nitrogen, organic carbon, available phosphorus and available potassium all increased with the increasing application rate. Total nitrogen content increased by 6.80–64.54% compared with CK. Under T3 treatment, the organic carbon content in the 0–10 cm and 20–40 cm soil layers increased by 32.75% (*p* < 0.05) and 76.85% (*p* < 0.05) compared with CK, respectively. The response of available phosphorus to the amendment was particularly pronounced. In the 20–40 cm soil layer, the T2 and T3 treatments increased about 5.65 times and 8.55 times compared with CK, respectively. The content of available potassium also showed a progressive increase, and each soil layer increased by 1.60–35.54% compared with CK. In contrast, the difference between EC treatments was not significant, with a slight rebound under high application rates.

Table A1 indicates the effects of different treatment amendment dosages on the composition of soil salt ions in each soil layer, mainly manifested by the decrease in Na^+^ and the increase in Ca^2+^. With the increasing calcium lactate treatment, the Na^+^ content in each soil layer decreased significantly. Specifically, T3 treatment had the most significant effect, decreasing by 89.61%, 80.43% and 62.50% compared with CK in 0–10 cm, 10–20 cm and 20–40 cm soil layers, respectively. Furthermore, most treatments reached significance (*p* < 0.05). Meanwhile, the Ca^2+^ content increased significantly, the increase in each soil layer reached 18.75–92.31%, and most treatments reached extreme significance (*p* < 0.001). Therefore, calcium lactate can promote desalination and sodium reduction by supplementing Ca^2+^ and replacing Na^+^ adsorbed by soil colloids. The Mg^2+^ content increased first and then decreased in general. The K^+^ content increased by 2.50–58.62% in T3 treatment compared with CK, while it failed to change significantly. In terms of anion, the SO_4_^2−^ content was the highest and no clear trend was observed. The SO_4_^2−^ content of T2 treatment was generally lower, decreasing by 13.40%, 27.59% and 10.00% compared with CK, respectively. The SO_4_^2−^ content of other treatments increased. The Cl- content decreased and then increased with the treatment from T1 to T3, but the content was lower than that of CK overall. The treatment with T1 produced the greatest improvement, with a decrease of 17.39–34.85%. The overall CO_3_^2−^ + HCO_3_^−^ content decreased slightly, with the largest decrease of 3.49–9.18% in the T2 treatment.

### 2.2. Dose Effects of Calcium Lactate Amendment on Growth, Yield and Quality of Pepper

**Figure 2:**
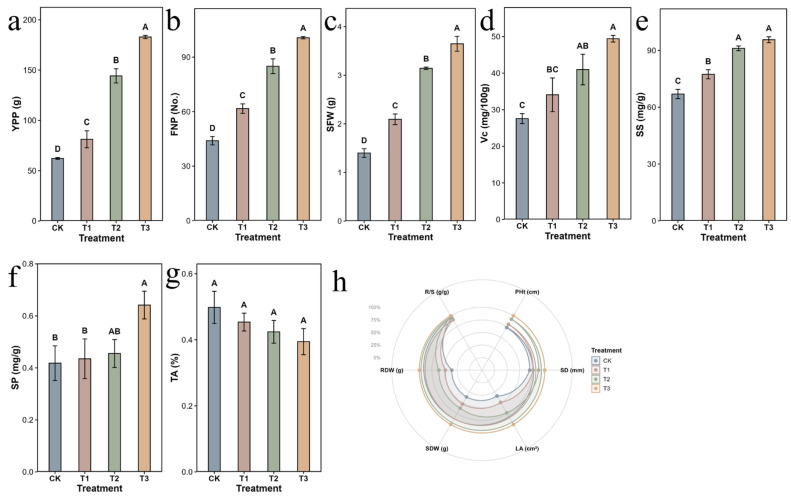
Effects of different dosages of calcium lactate amendments on the growth, yield and fruit quality of pepper. (**a**) Yield per plant (YPP); (**b**) fruit number per plant (FNP); (**c**) single fruit fresh weight (SFW); (**d**) vitamin C content (Vc); (**e**) soluble sugar content (SS); (**f**) soluble protein content (SP); (**g**) titratable acid content (TA); (**h**) radar chart of growth morphology indicators of pepper, including plant height (PHt), stem diameter (SD), leaf area (LA), shoot dry weight (SDW), root dry weight (RDW), and root–shoot ratio (R/S). Data are mean ± SD (*n* = 3), with different letters indicating significant differences between treatments (*p* < 0.05).

The application of calcium lactate significantly promotes the growth of pepper, and improves the yield and fruit quality, among which the effect of medium- and high-dose treatment is the most significant. According to Figure 2a–c, among all treatments, the T3 treatment achieved the highest values in terms of yield composition and stability. The weight per fruit, yield per plant and fruit number per plant of pepper all increased with the increasing amount of amendment. According to Figure 2d–g, the application of the amendment can significantly improve the quality attributes of the pepper fruit, which showed a clear dose-dependent pattern. Specifically, from T1 to T3 treatment, the contents of vitamin C, soluble sugar and soluble protein increased with the increasing amount of amendment, while the content of titratable acid presented a decreasing trend. For vitamin C, T1, T2 and T3 increased it by 23.58%, 48.64% and 79.17% compared with CK, and T3 treatment was significantly higher than other treatments. For soluble sugar, the content of the T3 treatment was the highest, significantly increasing by 42.80% compared with CK control, while the content of the T1 and T2 treatments was increased by 15.58% and 36.04% compared with CK. There was an extremely significant difference between T2 and T3 and CK (*p* < 0.001), which indicates that the ameliorant has a significant promoting effect on sugar accumulation. As for soluble protein, T3 treatment increased it by 53.41% compared with the control and had the best improvement effect. Regarding titratable acid, although there is no significant difference between the treatments, it exhibits a decreasing trend with the increasing amendment dosage. These results suggest that calcium lactate may improve fruit flavor through the regulation of fruit biochemical composition.

To comprehensively evaluate the effects of different treatments on pepper growth, a radar plot was constructed after standardizing growth indicators such as plant height (PHt), stem diameter (SD), leaf area (LA), shoot dry weight (SDW), root dry weight (RDW) and root–shoot ratio (R/S) (Figure 2h). The results indicate that the overall coverage area of different treatments is T3 > T2 > T1 > CK. Thus, the comprehensive growth performance of pepper was enhanced with the increase in amendment application. Specifically, T3 treatment was the highest in PHt, SD, LA, SDW and RDW, which formed the largest radar coverage area. Consequently, it significantly promotes the shoot growth and root development of plants.

### 2.3. Dose Effects of Calcium Lactate Amendment on Waterlogging Tolerance of Pepper

Calcium lactate significantly enhances the antioxidant defense of pepper, alleviates the oxidative damage caused by waterlogging stress, and improves plant stress resistance.

**Figure 3:**
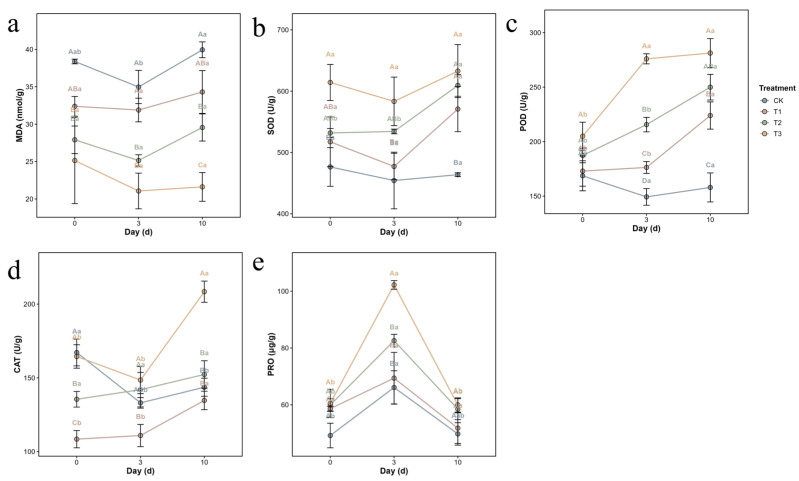
Effects of different dosages of calcium lactate amendments on the physiological indexes of waterlogging tolerance of pepper. (**a**) Malondialdehyde (MDA); (**b**) superoxide dismutase (SOD); (**c**) peroxidase (POD); (**d**) catalase (CAT); (**e**) proline (PRO). DAY 0, DAY 3, and DAY 10 represent the measurement periods before flooding, 3 days after flooding, and 7 days after recovery, respectively. The data are mean ± standard deviation (*n* = 3). Different uppercase letters indicate significant differences between various treatments at the same time (*p* < 0.05), and different lowercase letters denote significant differences between various treatments at different times (*p* < 0.05).

Figure 3a indicates that CK had the highest MDA content at any period, and the MDA content decreased with increasing amounts of amendment. Compared with the pre-waterlogging period, MDA of each treatment decreased by 1.54–16.22% on the third day of waterlogging, and T3 had the largest decrease. T1, T2 and T3 treatments decreased by 0.08-fold, 0.28-fold and 0.39-fold, respectively. After seven days of recovery, the MDA of all treatments except T3 increased from the initial level, and only the T3 treatment decreased by 14.04%. According to Figure 3b–d, calcium lactate significantly increased antioxidant enzyme activity and overall increased with increasing calcium lactate application. Before waterlogging, the activity of SOD increased by 8.61–28.95% compared with that of CK. Although the enzyme activity of each treatment decreased on the third day of waterlogging, it was still higher than that of CK. After seven days of recovery, SOD activity was further increased by 8.48–19.63%. The activity of POD increased continuously with the increasing application rate. T1, T2 and T3 increased by 18.08%, 44.42% and 84.82%, respectively, compared with CK in the waterlogging period. In the recovery period, except CK decreasing by 6.72%, each treatment still increased by 28.69–37.02% compared with that before waterlogging. The overall activity of CAT is characterized by fluctuation in the stress period and rebound in the recovery period. T1 and T2 increased by 2.27% and 4.80%, respectively, in the waterlogging period compared with the pre-waterlogging period, while T3 decreased by 9.72%. After 7 d of recovery (DAY10), all treatments showed significant recovery. Compared with the pre-waterlogging period, T1, T2 and T3 increased by 0.24-fold, 0.12-fold and 0.27-fold, respectively, among which T3 reached the highest. Proline content accumulated significantly during stress and decreased during the recovery period (Figure 3e). Compared with CK, calcium lactate treatment increased the proline content in a dose-dependent manner, among which T3 treatment had the most significant effect. It increased by 68.94% on the third day of waterlogging compared with the pre-waterlogging period, and 54.62% compared with CK in the same period (*p* < 0.05). In the recovery period, the proline content of each treatment dropped back.

**Figure 4:**
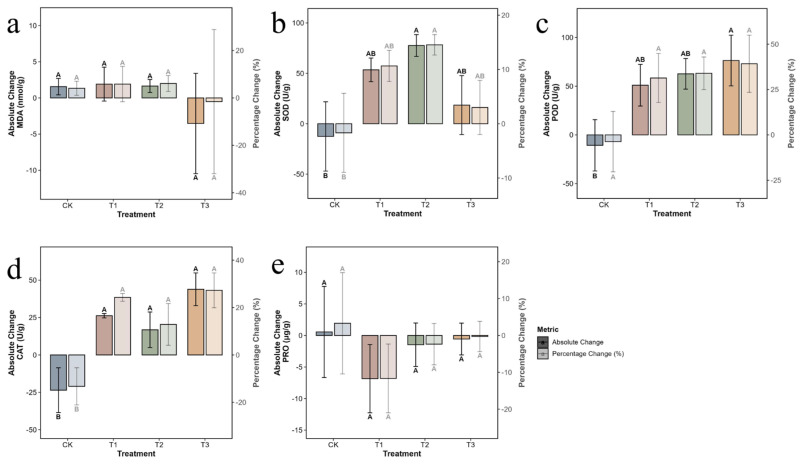
Changes of physiological indexes of waterlogging tolerance of pepper under different dosages of calcium lactate ameliorator on day 0 and day 10 during flooding treatment. (**a**) MDA; (**b**) SOD; (**c**) POD; (**d**) CAT; (**e**) PRO. The histogram represents the absolute amount of change and the relative rate of change of each index, respectively. DAY 0 and DAY 10 refer to the measurement period before flooding and 7 days after recovery, respectively. Data are mean ± SD (*n* = 3), with different letters indicating significant differences between treatments (*p* < 0.05).

Figure 4a–e reflect the changes in MDA, SOD, POD, CAT and proline in pepper leaves by different treatments before waterlogging (DAY0) and after 7 days of recovery (DAY10). In the recovery period, SOD, POD and CAT all presented an increasing trend in different degrees under the treatment of various modifiers, among which the increasing amplitude of T2 and T3 treatment was more apparent, while the overall showed a decreasing trend under CK treatment. MDA content presented an upward trend in CK, but exhibited a decrease or stabilization in T3 treatment. The proline content increased slightly in CK, but the change amplitude was small or even decreased under the amendment treatment.

### 2.4. Correlation Analysis Between Soil Environmental Factors and Growth, Yield, Quality and Waterlogging Tolerance of Pepper

**Figure 5:**
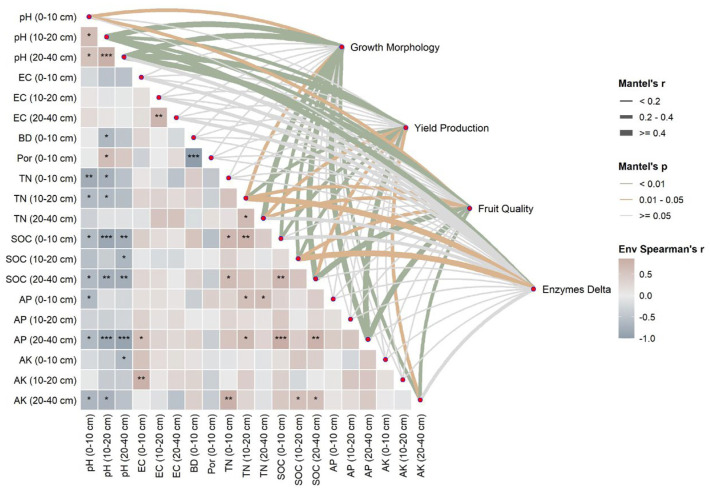
Correlation analysis between soil environmental factors and growth, yield, quality and waterlogging tolerance indexes of pepper. Mantel test and Spearman correlation analysis between soil environmental factors and plant response indicators. The thickness of the line indicates the size of Mantel’s r value, and the color of the line indicates the significance of the Mantel test (green: *p* < 0.01; orange: 0.01 ≤ *p* < 0.05; grey: *p* ≥ 0.05). Heat map color indicates the Spearman correlation coefficient among environmental factors. Blue and red refer to negative and positive correlation, respectively. *, **, and *** indicate significance at *p* < 0.05, *p* < 0.01, and *p* < 0.001, respectively. The modules of growth morphology, yield, fruit quality and stress resistance index change are included.

There is a significant correlation between soil physicochemical properties and growth, yield and stress resistance of pepper, which indicates that the soil environment is an essential driving factor to regulate the comprehensive response of plants. According to the results of the Mantel test, there was a significant coupling relationship between soil physicochemical properties and the growth, yield, quality and antioxidant enzyme changes of pepper (Figure 5). Overall, soil environmental factors were significantly correlated with growth morphology, yield formation and fruit quality (*p* < 0.05), and the correlation intensity varied. Specifically, pH, organic carbon (SOC) and total nitrogen (TN) presented high Mantel’s r values with each plant response module, and exhibited a stable multi-module connection relationship. Therefore, it plays a key role in driving the comprehensive phenotypic response of plants. Available phosphorus (AP) and available potassium (AK) indicate moderate and weak correlations with growth morphology, yield and quality indexes, which have auxiliary regulatory effects on plant response. Spearman correlation analysis further proves a significant positive correlation between SOC, TN, AP and AK, while there is a significant negative correlation between bulk density and porosity, which collectively form a stable synergistic network structure of soil physicochemical properties. There was also a significant negative correlation between pH and nutrient indexes such as SOC and AP, but the correlation intensity fluctuated in different soil layers. The overall results reveal the mechanism of soil nutrients and structural factors synergistically driving plant growth and stress resistance response.

**Figure A1:**
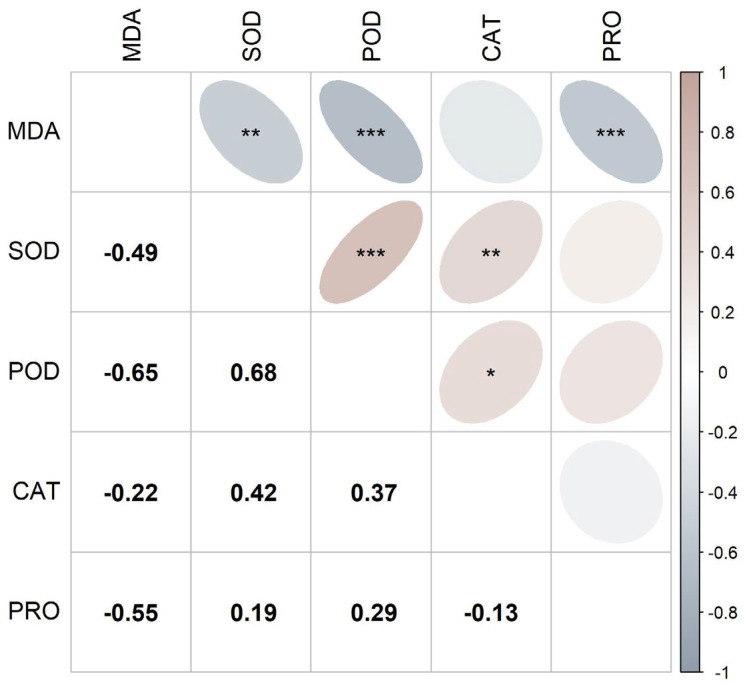
Spearman correlation analysis among physiological indexes of waterlogging tolerance in pepper. The color of the ellipse indicates the direction and intensity of the correlation; blue indicates negative correlation; red indicates positive correlation. The numbers in the figure represent Spearman’s correlation coefficient (r). *, **, and *** indicate significance at *p* < 0.05, *p* < 0.01, and *p* < 0.001, respectively.

According to Figure A1, MDA had a significant negative correlation with SOD, POD and proline contents, among which the negative correlation with POD was the strongest (r = −0.65, *p* < 0.001). Thus, the enhancement of protective enzyme activity and the accumulation of osmotic regulatory substances can effectively reduce membrane lipid peroxidation damage. Meanwhile, SOD showed significant positive correlation with POD (r = 0.68, *p* < 0.001) and CAT (r = 0.42, *p* < 0.01). Thus, the antioxidant enzyme system had an apparent synergistic effect in the process of reactive oxygen species scavenging. The above results demonstrate that calcium lactate treatment increases the tolerance of pepper to waterlogging stress by activating the antioxidant defense system and promoting the accumulation of osmotic-regulating substances.

### 2.5. Mechanism of Impact of Calcium Lactate Amendment, Soil Environment, Stress Resistance and Pepper Production Performance

**Figure 6:**
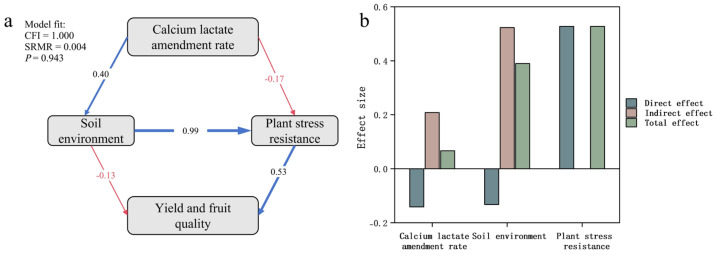
(**a**) PLS-PM model reveals the mechanism of impact of calcium lactate amendment on soil environment, Plant stress resistance and yield and quality of pepper. The rectangular box indicates the latent variable, the arrow indicates the path of action between the variables, and the number next to the arrow is the normalized path coefficient (β). The blue arrow refers to positive action and the red arrow indicates negative action. (**b**) Soil environment is characterized by soil physicochemical properties and nutrient indexes; plant stress resistance is characterized by antioxidant enzyme activity and oxidative damage indexes; yield and quality are characterized by yield and fruit quality indexes.

Calcium lactate may indirectly promote the yield and quality of pepper indirectly by improving the soil environment and strengthening plant stress resistance, in which the soil environment plays a key intermediary role. A partial least squares path model (PLS-PM) was used to analyze the indirect influence and direct relationship between calcium lactate amendment dosage, soil environment, plant stress resistance and pepper yield and quality (Figure 6). The model fit well (CFI = 1.000, SRMR = 0.004, *p* = 0.943), which can explain the mechanism of impact between latent variables. The results indicate that the dosage of calcium lactate amendment significantly promotes the improvement of soil environment (β = 0.40, *p* = 0.009), and the soil environment further significantly enhances plant stress resistance (β = 0.99, *p* < 0.001). In contrast, the direct effect of amendment dosage on plant stress resistance is weak and negative (β = −0.17, *p* = 0.008). The indirect effects generated through the soil environment are significantly higher than the direct effects, which indicates that improved soil conditions are the main driving factor in enhancing the stress resistance of peppers. Plant stress resistance further significantly promotes yield and quality improvement (β = 0.53, *p* = 0.185), while the direct effect of soil environment on yield and quality is weak (β = −0.13, *p* = 0.738). Generally, calcium lactate amendment mainly improves the yield and quality of pepper indirectly by improving the soil environment, thus enhancing plant stress resistance.

Overall, the application of calcium lactate significantly improves the physicochemical properties of soil, including nutrient availability and soil structure, while regulating the salt ion balance between soil layers. These improvements in the soil environment are accompanied by the improvement of growth, yield and fruit quality of pepper plants. In addition, calcium lactate significantly enhances the antioxidant defense system of plants and alleviates the oxidative stress caused by waterlogging. Correlation and Mantel analysis further prove a strong correlation between soil environmental factors and plant responses. Finally, structural equation model analysis revealed that the effects of calcium lactate on pepper performance may be primarily mediated through improvements in the soil environment, which subsequently regulate plant stress responses. This finding provides a potential pathway for understanding the regulation of saline soil–pepper system responses by calcium lactate; however, the specific causal relationships require further validation through long-term and multi-scale studies.

## 3. Discussion

### 3.1. Physiological Mechanism of Calcium Lactate Regulating Saline Soil Microenvironment and Promoting Plant Stress Resistance

Calcium lactate application significantly improves the physicochemical properties of salinized soil. Soil pH decreases with increasing application rate, which is consistent with the findings of Fan et al. [16]. The mechanism is that lactate radicals are degraded by microbes to produce acid and neutralize alkalinity. Meanwhile, Ca^2+^ replaces Na^+^ and promotes its elution [7,16], so that EC is significantly reduced at medium and low dosages. In this study, Na^+^ content decreased significantly with increasing calcium lactate application rates, whereas EC remained relatively stable, indicating that calcium lactate primarily altered soil salt ion composition rather than simply reducing total soluble salt content. This may be attributed to the fact that Ca^2+^ entering the soil system partially replenishes cation concentrations in the soil solution, thereby buffering changes in EC to some extent. However, EC slightly increased under the high application rate due to the introduction of soluble salts, suggesting the existence of an optimal amendment rate. Moreover, previous studies have shown that calcium-based amendments can promote soil organic carbon accumulation and improve soil physicochemical quality by regulating soil microbial community structure [29]. In this study, SOC content increased with increasing calcium lactate application rates, indicating that calcium lactate application may promote soil organic carbon accumulation; however, its specific mechanism requires further validation by integrating changes in microbial community structure and carbon fractions. Moreover, Ca^2+^ enhanced the stability of aggregates by compressing the electric double layer and promoting clay flocculation [30], which significantly reduced the soil bulk density and increased the porosity. In this study, Na^+^ decreased significantly, while Ca^2+^ continued to increase, which proves that the Ca^2+^-dominated ion exchange process is the key to desalination. Cl^−^ and CO_3_^2−^ + HCO_3_^−^ decreased overall. Consequently, calcium lactate contributes to a reduction in basic anion accumulation. Gypsum can provide Ca^2+^ ions in soil solution, accompanied by SO_4_^2−^ as a relatively stable anion [31], but the change of SO_4_^2−^ in this experiment is not apparent. As a result, lactate can be degraded by microorganisms without an obvious anion residue, which is also the difference between calcium lactate and traditional gypsum-based amendments, thus reducing the long-term accumulation of inorganic anions in soil. The improvement of the soil nutrient pool by calcium lactate may result from its integrated regulation of the soil microenvironment. Calcium lactate provides readily available carbon and calcium sources, which may improve the rhizosphere environment and promote soil nutrient transformation processes. In this study, SOC, TN, AP, and AK contents increased with increasing calcium lactate application rates, indicating that calcium lactate improved nutrient availability in saline soil. Moreover, the improvement of pH and the protection of organic carbon by Ca^2+^-bridged aggregates jointly enhanced carbon utilization efficiency. The several-fold increase in available phosphorus in deeper soil layers may be related to the improvement of soil chemical conditions and enhanced phosphorus migration induced by calcium lactate. Soil pH is a key factor affecting phosphorus availability, as phosphorus can easily combine with Ca^2+^ under high-pH conditions to form insoluble calcium phosphate, thereby reducing available phosphorus content [32]. Therefore, reduced pH may decrease phosphorus fixation in saline soil and improve phosphorus availability; meanwhile, improved soil structure enhances water movement and nutrient transport capacity [33], facilitating the migration of soluble phosphorus to deeper soil layers. Although Ca^2+^ may form stable Ca-P minerals with phosphorus [34], its fixation effect may be partially offset by improvements in the soil environment induced by calcium lactate. The increase in available potassium may be attributed to enhanced potassium release and migration following the reduction in soil bulk density. Calcium lactate integrates multiple functions, including alkalinity reduction, carbon enrichment, and nutrient activation, providing a new approach for the sustainable improvement of saline–alkali soils.

Short-term waterlogging stress restricts oxygen diffusion in the rhizosphere, inhibits plant respiration, and induces excessive accumulation of reactive oxygen species (ROS), thereby resulting in membrane lipid peroxidation [35]. The study indicates that the MDA content continues to decrease with the increase in calcium lactate application, and the T3 treatment in the recovery period is still lower than the pre-stress period. Thus, calcium lactate enhances membrane stability while reducing oxidative damage with a sustained protective effect. After calcium lactate is applied, the activities of SOD, POD and CAT in each treatment are significantly increased, which demonstrates that the modifier strengthens the antioxidant defense, effectively removes ROS and alleviates stress. Proline, as an essential osmotic regulating substance, further enhances the stress resistance of plants by maintaining cell bulking pressure and stabilizing protein structure [36]. Proline content increases rapidly with the increase in calcium lactate dosage, while calcium lactate treatment decreases to pre-stress levels faster during the recovery period. Thus, calcium lactate can promote osmotic regulation and accelerate metabolic recovery [13]. Similarly, Chen et al. [37] found that environmental regulation materials promoted plant adaptation to stress by enhancing antioxidant enzyme activities and reducing oxidative damage, which was consistent with the enhanced antioxidant defense induced by calcium lactate in this study, suggesting that maintaining plant redox homeostasis may be an important mechanism by which different regulatory strategies promote plant stress adaptation. Overall, amendment treatment can significantly alleviate the accumulation of oxidative damage indexes after flooding stress, and enhance the recovery of antioxidant enzyme activity. This study systematically reveals the physiological basis of calcium lactate to alleviate waterlogging stress from three aspects of membrane lipid peroxidation, antioxidant defense and osmotic regulation, which provides a theoretical basis for calcium lactate to strengthen the stress resistance of pepper in salinized facility soil.

According to the results of correlation analysis, there is a strong correlation between soil fertility and the yield and quality indexes of pepper. It shows that crop response is not driven by a single factor, but coordinated by soil physicochemical properties and nutrient network. Specifically, pH, SOC and TN present a high overall correlation strength and maintain a stable role in multiple functional modules, which indicates that they have a fundamental driving role in maintaining soil nutrient supply and system stability. This result is consistent with the previous conclusion that “soil organic carbon and nitrogen cycles determine ecosystem productivity” [38,39]. In contrast, AP and AK only exhibit a moderate or weak correlation. Consequently, AP mainly participates in short-term nutrient regulation, rather than being the dominant limiting factor. Further analysis proves a significant positive correlation network between SOC, TN, AP and AK, while BD and Por show a significant negative correlation. Thus, soil nutrients and structural factors jointly constitute a stable but hierarchically different soil fertility system. The underlying mechanism is that the application of soil amendment systematically modifies soil pH, bulk density, porosity, available potassium, available phosphorus and other contents, so as to optimize soil physical structure and chemical fertility simultaneously. On the one hand, the modifier directly reduces the bulk density, increases the porosity, alleviates the mechanical stress of the root system and improves the aeration and water permeability. On the other hand, it enhances nutrient storage capacity and nutrient availability, providing a nutrient basis for plant growth and development and fruit quality formation. More importantly, the improvement of soil environment creates a favorable growth environment for microbial growth, and may enrich beneficial microbial taxa [40], which in turn enhances crop tolerance to saline–alkali stress and promotes plant growth and fruit quality formation.

According to the results of plant physiological response, MDA is significantly negatively correlated with SOD, POD and PRO, among which POD is the most prominent. Consequently, the antioxidant system plays a key role in alleviating membrane lipid peroxidation damage [41]. Meanwhile, the significant positive correlation between SOD, POD and CAT indicates that the antioxidant system has a synergistic effect, while PRO enhances the stress resistance through osmotic regulation. This synergistic effect ensures the efficient clearance of ROS and prevents the destruction of cellular structure by oxidative stress [42,43]. On the whole, calcium lactate improvement jointly drives the growth, yield and waterlogging tolerance of pepper by reconstructing soil nutrient structure and physical environment, and activating the plant antioxidant defense system, which forms a synergistic regulatory mechanism between soil and plants.

### 3.2. Potential Pathways of Calcium Lactate Regulating Pepper Growth and Quality Formation

In this study, the partial least squares path model (PLS-PM) is used to explore the potential pathways through which calcium lactate amendment affects pepper growth and quality formation. The results showed that calcium lactate application rates mainly influenced plant stress responses by improving the soil environment, and this pathway may be involved in the growth and quality formation of pepper. This mechanism is consistent with the research of Wu et al. (2024) [44] on microbial fertilizers to enhance crop stress resistance by regulating soil microecology, and jointly emphasizes the core position of the rhizosphere environment as a regulatory hub. Previous studies have shown that good soil structure and nutrient supply can promote root vitality and maintain ionic balance and energy metabolism, thus enhancing antioxidant defense system function [45]. The increased activities of SOD, POD, and CAT and the decrease in MDA content in this study further support this mechanism that calcium lactate does not function solely as a calcium source, but indirectly enhances the adaptability of plants to waterlogging stress by improving the soil environment.

Notably, although the path relationship between stress resistance indicators and yield and quality was positive (β = 0.53), it did not reach statistical significance (*p* = 0.185), indicating that this relationship requires further validation through larger sample sizes and long-term experiments. Therefore, the PLS-PM results in this study were mainly used to reveal potential associations among variables rather than to demonstrate definitive causal mechanisms. The direct pathway from amendment dosage to plant stress resistance was negative (β = −0.17), which may reflect the influence of soil ionic environment changes caused by high-dose calcium lactate input on plant responses, whereas the positive effects of calcium lactate were mainly manifested through indirect effects after soil environment improvement. This result suggests that the effects of calcium lactate are jointly influenced by application rate and soil environmental conditions. This finding further indicates that focusing solely on plant physiological responses may underestimate the importance of soil improvement processes in waterlogged facility soils. Compared with short-term physiological regulation, soil environment optimization can continuously improve root growth conditions, thereby supporting enhanced plant stress resistance and production performance.

Overall, this study constructs a potential action framework of calcium lactate amendment on pepper production performance, revealing that calcium lactate may enhance pepper growth, yield, and quality by improving the saline soil environment, optimizing soil structure, and strengthening plant stress responses, and provides a new theoretical basis for the application of calcium-based amendments in saline soil management. As an environmentally friendly calcium-based amendment, calcium lactate shows considerable application potential in waterlogged salinization facility soil. It can achieve a synergistic effect of improving quality and yield by reducing alkali and desalination, improving soil structure, and enhancing nutrient availability and crop stress resistance, which is suitable for saline–alkali land and facility vegetable production systems. Compared with previous studies on calcium lactate amendment in saline–alkali soils [16], this study further focused on crop responses under short-term waterlogging events during agricultural production in saline soils and revealed the synergistic regulation of soil environment and plant stress resistance by calcium lactate. However, this study has several limitations. Since all treatments were conducted under short-term waterlogging conditions, a non-waterlogged control was not included. Therefore, this experiment mainly evaluated the effects of different calcium lactate application rates on saline soil environments and pepper growth responses under waterlogging conditions, rather than completely distinguishing the relative contributions of soil amendment effects and plant adaptation to waterlogging. Moreover, this study is only based on a single season, single soil and crop conditions. The long-term effect of high doses and microbial driving mechanisms remains to be validated, and the model index system has limitations. In the future, it is necessary to carry out long-term multi-site experiments to analyze soil and rhizosphere microbial processes combining metagenomes and metabolomes, and evaluate the synergistic effects and environmental impacts with other amendments, so as to optimize application strategies and improve their theory and application system in green agriculture.

## 4. Materials and Methods

### 4.1. Overview of the Study Area

Jinxiang County, under the jurisdiction of Jining City, Shandong Province, is located in the southwest of Shandong Province, with a total area of 886 square kilometers. There are gentle slopes and depressions in the county, which belongs to a warm temperate monsoon continental climate with an average annual precipitation of 726.19 mm. In summer, the precipitation is concentrated with many heavy rains, and the low-lying areas are prone to waterlogging during heavy precipitation. The cultivated land is mainly fluvo-aqueous soil with loose soil quality, which is suitable for cultivating crops. Jinxiang pepper is one of the famous local specialties, and Jinxiang is also known as “the hometown of Chinese pepper”, with a pepper planting area of 420,000 mu (a Chinese unit of area).

### 4.2. Test Design

The test plot is in Houzhou Village, Jinxiang County, Jining City, Shandong Province (116°15′29.73″ E, 35°11′1.07″ N). The tested pepper variety is “Sanying No. 8”, and the tested soil is saline Fluvisol (alluvial soil) in Jinxiang, Jining. The basic physicochemical properties of the soil at the 0–10, 10–20, and 20–40 cm soil depths are presented in Table A1. The test material is a new modifier with calcium lactate as the main component. In this test, the dosage of calcium lactate modifier is a variable, and four treatments are set up: CK (no calcium lactate, waterlogged control), T1 (low-dose calcium lactate, 600 kg·hm^−2^), T2 (medium-dose calcium lactate, 1200 kg·hm^−2^), and T3 (high-dose calcium lactate, 2400 kg·hm^−2^). Four plots were established in the experiment, each plot had three replicates, and soil samples were collected before the application of the amendment, during the waterlogging period and during the pepper harvesting period. Soil samples of 0–10 cm, 10–20 cm and 20–40 cm soil layers were collected in each plot by the five-point sampling method, and the soil samples of the same layer in the same plot were evenly mixed with a total of 36 samples. After removing gravel and plant residues, the soil samples were taken to the air drying room for natural air drying and the soil samples were ground with a grinder, and sieved according to the measurement indicators to be tested (the samples of each index are measured three times).

In this test, double planting of garlic-set pepper was conducted in 2025, with a spacing of 18 cm between plants and 72 cm between rows. Irrigation was carried out on the day of transplantation, and irrigation was subsequently applied at two-week intervals, with a water volume of about 20–30 mm. Five days after garlic harvesting, the amendment was evenly incorporated into the soil into a 20 cm deep ditch according to the test scheme, covered with soil and mixed evenly, so that it is fully contacted with the tillage soil. Except for the calcium lactate treatments, all fertilization, irrigation, and field management practices were kept consistent among experimental plots to ensure identical nutrient inputs among different treatments. During garlic cultivation, compound fertilizer (15-15-15) was applied at a rate of 2250 kg·hm^−2^, providing 337.50 kg·hm^−2^ of N, P, and K, respectively. During the flowering and fruit-setting stage of pepper, topdressing with compound fertilizer (18-12-20) was applied at a rate of 450 kg·hm^−2^, providing 81, 54, and 90 kg·hm^−2^ of N, P, and K, respectively. The 3-day flooding duration was determined based on the characteristics of short-term water accumulation in low-lying farmland following concentrated summer rainfall in the local region, and was used to simulate sudden waterlogging stress during protected pepper production. Waterlogging stress was simulated by artificial flooding, and the experiment set CK and three treatments. The indexes are also measured at 0 d (DAY0), 3 d (DAY3) and 7 d (DAY10) after flooding. The test is controlled by pipelines and valves, so that the water layer in the community is kept 3 cm higher than the ground surface. During the test, water is replenished regularly in the morning, midday and evening every day to keep the water level constant.

**Figure 7:**
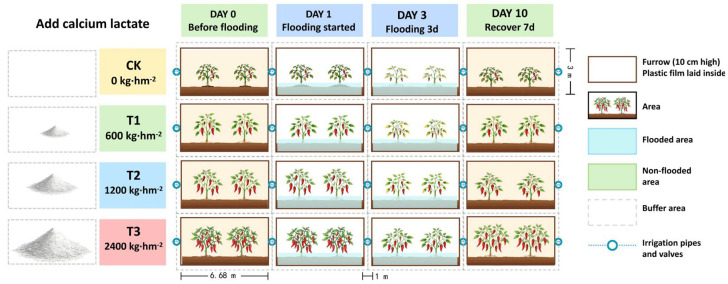
Schematic diagram for the test design of the calcium lactate amendment treatment and waterlogging stress. Four treatments are established: blank control (CK, 0 kg·hm^−2^), low-dose calcium lactate treatment (T1, 600 kg·hm^−2^), medium-dose calcium lactate treatment (T2, 1200 kg·hm^−2^), and high-dose calcium lactate treatment (T3, 2400 kg·hm^−2^). Calcium lactate is applied uniformly to the soil before the start of the experiment. Day 0 represents the initial status before waterlogging treatment. Waterlogging stress is simulated by maintaining 3 cm of standing water on days 1 to 3. As for day 10, after 3 days of flooding, the flooding is stopped and normal management is restored for 7 days. The figure indicates the growth state changes of pepper plants under various treatments during the occurrence and recovery of waterlogging. The area of each experimental plot is 6.68 m × 3.0 m.

The experiment followed a randomized complete block design with three replicates per treatment for 12 cells in total. The subdivision is 6.68 m long and 3 m wide, with an area of about 20 m^2^. A 1 m isolation belt is set between the residential areas, and a 10 cm high ridge is built around it with plastic film to prevent lateral seepage and water (Figure 7). Pipeline interfaces are reserved, and the irrigation amount of each residential area is controlled through independent valves to ensure accurate and consistent waterlogging treatment. Other agronomic management during the test is conducted based on local routine.

### 4.3. Analysis of Soil Physicochemical Properties

Basic physicochemical indexes as well as nutrient indexes of soil are measured by conventional analytical methods. Soil pH is measured using a soil suspension with a water-to-soil ratio of 2.5:1 using a pH meter (PHSJ-4A, Shanghai Leici, Shanghai, China). Soil electrical conductivity (EC) is measured by a conductivity meter (DDS-11A, Shanghai Leici) on soil suspension with a water-to-soil ratio of 5:1. Soil bulk density and porosity are determined using the core ring method after soil samples are collected and oven-dried. The contents of K^+^, Na^+^, Ca^2+^ and Mg^2+^ in soil leachate (water to soil ratio 5:1) are measured by flame atomic absorption spectrometry. Cl^−^ is titrated by silver nitrate. SO_4_^2−^ is titrated by EDTA indirect complexation. HCO_3_^−^ and CO_3_^2−^ are titrated using a dual indicator neutralization. Organic carbon and total nitrogen are measured on-machine using the elemental analyzer (Thermo Fisher Scientific, Waltham, MA, USA). The available phosphorus is extracted by the 0.5 mol·L^−1^ NaHCO_3_^−^ molybdenum antimony colorimetric method. The available potassium is extracted by NH_4_OAc-flame spectrophotometry. In addition, the measurement of each index is repeated three times.

### 4.4. Sample Collection and Measurement of Pepper Plants

Three representative plants are randomly selected from each plot during the harvesting period of pepper. Plant height and stem diameter are measured by a tape measure and an electronic vernier caliper, respectively. The leaf area is measured by the YMJ-B leaf area meter at the third leaf from the top to the bottom. For biomass measurement, the pepper plants are washed and heated at 105 °C for 30 min to deactivate enzymes, then dried to constant weight at 80 °C, and the dry mass is weighed to calculate the root-to-shoot ratio (the ratio of the root system to the above-ground mass of the plant). The fruits of the selected plants are picked, and the weight per fruit, the number of fruits per plant and the yield per plant are measured, respectively. The fresh leaves of pepper plants are ground into a homogenate. Then, the soluble sugar, vitamin C and soluble protein are measured by corresponding detection kits. Titratable acids are measured by 0.1 mol·L^−1^ NaOH titration. Fresh functional leaves are collected at a fixed time in each plot after the end of waterlogging stress and at the harvest period. The area of the same leaf at the picking site is also measured immediately. Meanwhile, 0.20 g of leaves is weighed, a certain ratio of phosphate buffer solution is added, and a homogenate solution is prepared under ice water bath conditions and centrifuged to measure the activities of malondialdehyde (MDA), superoxide dismutase (SOD), peroxidase (POD) and catalase (CAT), respectively, by corresponding kits. The content of proline (PRO) is homogenized from the leaves and measured using corresponding kits. Each of the above indicators is measured three times, and the kit is purchased from Nanjing Jiancheng Biological Co., Ltd. (Nanjing, China).

### 4.5. Data Analysis

#### 4.5.1. Descriptive Statistics

The trial data are collated and analyzed using Microsoft Excel 2019, with the results expressed as mean ± SD (*n* = 3).

#### 4.5.2. ANOVA

At each sampling time and soil depth, one-way analysis of variance (ANOVA) is performed separately to evaluate the differences among different treatments. Data analysis and statistics are performed using R 4.4.1. One-way ANOVA is also used to test the differences between various treatments, and multiple comparisons are performed using the least significant difference method (LSD), with *p* < 0.05 as the significance of difference and *p* < 0.001 as the extreme significance of difference. Data visualization and graphing are conducted using Origin 2024b.

#### 4.5.3. Correlation Analysis

Pearson correlation analysis aims to evaluate the correlation between soil physicochemical properties and plant stress resistance, yield and quality indexes. The correlation among variables is characterized by the correlation coefficient r. The Mantel test is used to analyze the overall correlation between soil environmental factors and plant growth, stress resistance, yield and quality indexes. In addition, the key soil factors affecting the growth, stress resistance, yield and quality of pepper are screened by visualization analysis through a correlation network diagram.

#### 4.5.4. Path Analysis

The partial least squares path model (PLS-PM) aims to investigate the mechanism of calcium lactate amendment affecting the yield and quality of pepper through the soil environment and plant stress resistance. In the model, the amount of amendment is used as the exogenous latent variable, and the soil environment, plant stress resistance, yield and quality are used as the endogenous latent variables. The significance of the path coefficient is tested, and the model’s explanatory ability is evaluated by the coefficient of determination (R^2^).

## 5. Conclusions

This study investigated the effects of calcium lactate amendment on the soil environment, plant stress resistance, yield, and fruit quality of pepper under waterlogging conditions. The results indicate that the application of calcium lactate can significantly improve the physicochemical properties of soil, reduce the pH and Na^+^ content of soil, increase the Ca^2+^ content, and promote soil desalination and alkali reduction. At the same time, the soil bulk density is reduced, the porosity is increased, and the nutrient contents, such as total nitrogen, organic carbon, available phosphorus and available potassium, are increased, thereby improving soil structure and nutrient availability. The enhancement of the soil environment promotes the root growth and leaf photosynthesis of pepper, improves the absorption and utilization efficiency of water and nutrients by plants, and significantly enhances fruit quality, including vitamin C, soluble sugar, and soluble protein contents, while increasing fruit number, fruit weight, and yield. Under waterlogging stress, calcium lactate enhances the activities of SOD, POD, and CAT, promotes proline accumulation, and reduces MDA content, thereby alleviating oxidative damage and improving plant stress resistance. Correlation analysis indicates that the yield and quality of pepper are mainly regulated by the synergistic network of soil nutrients and structure, with pH, SOC and TN as the core, while AP and AK are only secondary short-term regulatory factors. Bulk density and porosity affect the plant response by regulating the soil physical environment. PLS-PM analysis further suggests that calcium lactate may primarily influence plant stress responses by improving the soil environment. This result provides a potential pathway for understanding the regulation of saline soil–pepper system responses by calcium lactate; however, the causal relationships require further validation through future studies. Overall, calcium lactate enhances pepper productivity by improving soil conditions and strengthening plant stress resistance. Among all treatments, T3 exhibited the best overall performance, which can provide theoretical support and practical guidance for salinized soil improvement and efficient production of pepper in an open field.

## Data Availability

The original contributions presented in this study are included in the article. Further inquiries can be directed to the corresponding author.

## Appendix A

**Table A1:** Effect of different treatment amendments on the composition of soil salt segregant in different soil layers (×10^−2^ g·kg^−1^). Different lowercase letters indicate significant difference among treatments (*p* < 0.05).

Soil Depth (cm)	Treatment	K^+^	Na^+^	Ca^2+^	Mg^2+^	Cl^−^	SO_4_^2−^	CO_3_^2−^ + HCO_3_^−^
0–10	CK	1.05 ± 0.26 a	1.54 ± 0.81 a	0.13 ± 0.01 d	0.24 ± 0.05 b	0.77 ± 0.47 a	0.97 ± 0.07 a	0.65 ± 0.02 a
	T1	1.18 ± 0.23 a	0.57 ± 0.14 b	0.18 ± 0.02c	0.43 ± 0.01 a	0.63 ± 0.25 a	1.20 ± 0.31 a	0.63 ± 0.06 a
	T2	1.04 ± 0.09 a	0.36 ± 0.15 b	0.22 ± 0.00 b	0.30 ± 0.04 b	0.69 ± 0.06 a	0.84 ± 0.55 a	0.62 ± 0.04 a
	T3	1.37 ± 0.29 a	0.16 ± 0.10 b	0.25 ± 0.01 a	0.05 ± 0.08 b	0.72 ± 0.14 a	1.48 ± 2.18 a	0.62 ± 0.06 a
10–20	CK	0.87 ± 0.11 a	3.22 ± 1.17 a	0.16 ± 0.00 d	0.40 ± 0.09 a	0.69 ± 0.36 a	0.58 ± 0.42 a	0.06 ± 0.18 a
	T1	0.96 ± 0.29 a	1.96 ± 0.74 ab	0.19 ± 0.01 c	0.46 ± 0.09 a	0.57 ± 0.18 a	1.18 ± 0.95 a	0.84 ± 0.04 a
	T2	0.88 ± 0.34 a	0.94 ± 0.65 b	0.23 ± 0.01 b	0.45 ± 0.06 a	0.64 ± 0.36 a	0.42 ± 0.32 a	0.83 ± 0.08 a
	T3	0.93 ± 0.11 a	0.63 ± 0.20 b	0.26 ± 0.00 a	0.41 ± 0.10 a	0.66 ± 0.42 a	0.79 ± 0.40 a	0.84 ± 0.21 a
20–40	CK	0.64 ± 0.05 a	4.16 ± 1.21 a	0.16 ± 0.02 d	0.29 ± 0.04 c	0.66 ± 0.39 a	0.80 ± 0.46 a	0.98 ± 0.04 a
	T1	0.69 ± 0.20 a	3.82 ± 0.67 a	0.21 ± 0.01 c	0.57 ± 0.02 a	0.43 ± 0.15 a	1.18 ± 0.04 a	0.95 ± 0.17 a
	T2	0.60 ± 0.09 a	1.99 ± 0.56 b	0.24 ± 0.01 b	0.55 ± 0.06 a	0.52 ± 0.20 a	0.72 ± 0.68 a	0.89 ± 0.23 a
	T3	0.60 ± 0.23 a	1.56 ± 0.71 b	0.26 ± 0.01 a	0.46 ± 0.06 b	0.60 ± 0.05 a	1.50 ± 1.05 a	0.92 ± 0.09 a

**Table A2:** Basic physical and chemical properties of tested soil.

Soil Depth (cm)	pH	EC (mS·cm^−1^)	TC (g·kg^−1^)	TN (g·kg^−1^)	AP (mg·kg^−1^)	AK (mg·kg^−1^)
0–10	8.72	0.26	11.58	0.69	58.93	290.09
10–20	8.85	0.32	11.33	0.60	38.88	266.82
20–40	9.02	0.42	10.19	0.30	2.41	143.46
